# Milk exosomal miRNAs: potential drivers of AMPK-to-mTORC1 switching in β-cell de-differentiation of type 2 diabetes mellitus

**DOI:** 10.1186/s12986-019-0412-1

**Published:** 2019-12-06

**Authors:** Bodo C. Melnik

**Affiliations:** Department of Dermatology, Environmental Medicine and Health Theory, University of Osnabrück, Am Finkenhügel 7A, D-49076 Osnabrück, Germany

**Keywords:** AMP-activated protein kinase, Beta-cell de-differentiation, Beta-cell metabolic switch, Diabetes mellitus type 2, Estrogen-related receptor gamma, Exosome, miRNA-148a, Mechanistic target of rapamycin complex 1, Pasteurized milk, Weaning

## Abstract

Type 2 diabetes mellitus (T2DM) steadily increases in prevalence since the 1950’s, the period of widespread distribution of refrigerated pasteurized cow’s milk. Whereas breastfeeding protects against the development of T2DM in later life, accumulating epidemiological evidence underlines the role of cow’s milk consumption in T2DM. Recent studies in rodent models demonstrate that during the breastfeeding period pancreatic *β*-cells are metabolically immature and preferentially proliferate by activation of mechanistic target of rapamycin complex 1 (mTORC1) and suppression of AMP-activated protein kinase (AMPK). Weaning determines a metabolic switch of *β*-cells from a proliferating, immature phenotype with low insulin secretion to a differentiated mature phenotype with glucose-stimulated insulin secretion, less proliferation, reduced mTORC1- but increased AMPK activity. Translational evidence presented in this perspective implies for the first time that termination of milk miRNA transfer is the driver of this metabolic switch. miRNA-148a is a key inhibitor of AMPK and phosphatase and tensin homolog, crucial suppressors of mTORC1. *β*-Cells of diabetic patients return to the postnatal phenotype with high mTORC1 and low AMPK activity, explained by continuous transfer of bovine milk miRNAs to the human milk consumer. Bovine milk miRNA-148a apparently promotes *β*-cell de-differentiation to the immature mTORC1-high/AMPK-low phenotype with functional impairments in insulin secretion, increased mTORC1-driven endoplasmic reticulum stress, reduced autophagy and early *β*-cell apoptosis. In contrast to pasteurized cow’s milk, milk’s miRNAs are inactivated by bacterial fermentation, boiling and ultra-heat treatment and are missing in current infant formula. Persistent milk miRNA signaling adds a new perspective to the pathogenesis of T2DM and explains the protective role of breastfeeding but the diabetogenic effect of continued milk miRNA signaling by persistent consumption of pasteurized cow’s milk.

## Introduction

Milk, sugar and saturated fat are substantial components of Western diet. The per capita milk consumption in the United States and Germany in 2017 was 65.2 L and 52.2 L, respectively [1, 2]. An increasing number of epidemiological studies identified the consumption of commercial cow’s milk with an increased risk of insulin resistance and type 2 diabetes mellitus (T2DM) [3–9] (Table 1). In contrast, fermented milk products have been associated with a decreased risk of T2DM [10]. A nested case-cohort within 8 European countries of the European Prospective Investigation into Cancer and Nutrition (EPIC) Study (*n* = 340,234) analyzed the amount and type of dairy product intake and incident T2DM and identified an increased risk for T2DM by milk consumption in 5 of 8 countries [7]. The prospective Dutch Lifeline Cohort Study (*n* = 112,086) investigated the association of non-fermented milk products, milk and fermented milk products on participants with prediabetes (defined as fasting plasma glucose between 5.6 and 6.9 mmol/L or HbA1c of 5.7–6.4%) and newly diagnosed T2DM (defined as fasting plasma glucose > 7.0 mmol/L or HbA1c > 6.5%) [8]. A positive association between full-fat milk consumption (150 g/day) as well as non-fermented dairy products with prediabetes and T2DM has been shown [8]. Apparently, there is a diabetogenic ingredient in non-fermented milk compared to fermented milk products. A substantial difference between non-fermented pasteurized milk and fermented milk products is the fact that pasteurized milk contains bioactive miRNA-enriched exosomes and extracellular vesicles that survive degradation in the gastrointestinal tract [11–15]. Milk exosomes are taken up by endocytosis in intestinal and endothelial cells [16, 17], are bioavailable for human milk consumers [18], and reach the systemic circulation of the human milk consumer in a dose-dependent manner [19]. After oral administration, bovine milk exosomes are transported into various tissues and organs as recently demonstrated in several mouse models [20, 21]. Therefore, milk exosomes are regarded as promising new drug carriers to reach distant tissues for pharmacological intervention [22, 23]. Milk miRNAs including bovine miRNA-148a and miRNA-29b, which are identical to human miRNAs (mirbase.org), resist pasteurization, homogenization and refrigerated storage [24–26]. A recent study focusing on human skim milk identified 10 miRNAs that accounted for > 70% of the reads mapped to miRNAs. Among them miRNA-148a represented around 30% of the reads [27]. Notably, miRNA-148a is the most abundant miRNA in human and bovine milk fat and milk exosomes [25–29].

**Table 1:** Epidemiological studies showing a link between milk consumption, insulin resistance and type 2 diabetes mellitus

Study (R = retrospective; P = prospective)	n	Type of milk	Ref.
Dietary intervention milk vs. meat (P) Denmark	24	Skim milk	[3]
British Women’s Heart and Health Study (R) UK	4024	Skim, low-fat, whole milk	[4]
EPIC-Interact Study (P) Europe	340,234	Not fermented milk	[7]
Physicians´ Health Study (P) USA	21,660	Skim, low-fat, whole milk	[6]
Mendelian randomization study (R) Denmark	97,881	Milk, fat-free milk	[5]
Framingham Heart Study Offspring Cohort (P) USA	2809	Whole milk	[9]
Lifelines Cohort Study (P) Netherlands	112,086	Full-fat, skim milk	[8]

Accumulating evidence supports the role of milk-derived exosomal miRNAs in systemic metabolic regulation [30–38]. In contrast to pasteurized commercial milk, exosome integrity and miRNA content of fermented milk products such as yoghurt are degraded by bacterial fermentation [39]. The protein content of milk exosomes and their miRNA expression monitored by miRNA-29b and miRNA-21 were significantly reduced after fermentation [39]. Thus, commercial pasteurized milk in contrast to fermented milk products is a donor of bioactive exosomal miRNAs.

### Weaning triggers *β*-cell maturation

Recent evidence from murine models indicates that weaning, i.e., the termination of milk intake, triggers a critical maturation step of pancreatic *β*-cells [40, 41]. Weaning coincides with enhanced glucose-stimulated insulin secretion (GSIS) from islets [40]. Jaafar et al. recently demonstrated that a switch from the nutrient sensor *mechanistic target of rapamycin complex 1* (mTORC1) to the energy sensor *5′-adenosine monophosphate-activated protein kinase* (AMPK) is of critical importance for functional maturation of *β*-cells during weaning [41]. AMPK was activated by the dietary transition taking place during weaning, and this in turn inhibits mTORC1 activity to promote the adult *β*-cell phenotype [41]. Notably, milk has been characterized as a postnatal activator of mTORC1 for postnatal growth, translation and anabolism [42]. mTORC1 activation is required for the development and growth of *β*-cells during embryonic and early postnatal life [43–47]. AMPK is a potent inhibitor of mTORC1 activation [48, 49] and is the key target of the common antidiabetic drug metformin, which activates AMPK and inhibits mTORC1 [50, 51]. Jaafar et al. hypothesized that postnatal *β*-cell maturation may represent an adaptation to the cessation of milk consumption, and that mTORC1 repression through AMPK activation may act as a physiological mediator of this process [41]. Intriguingly, allowing mice to continue assimilating milk fat throughout their entry into adulthood, a period during which this is usually declining, was sufficient to allow *β*-cells to maintain neonatal levels of mTORC1 activity, which was otherwise completely repressed in milk-free control mice [41]. In addition, recent evidence supports the view that miRNAs play a key role in regulating *β*-cell differentiation and *β*-cell identity. A shift in miRNA expression has been associated with postnatal *β*-cell differentiation [52–57].

These observations lead to the three questions: 1) Is the cessation of milk-derived miRNAs the responsible mechanism for the metabolic switch of immature to differentiated *β*-cells? 2) Does continued bovine miRNA signaling by persistent consumption of cow’s milk induce the immature *β*-cell phenotype of T2DM? 3) Does miRNA-deficient infant formula feeding disturb appropriate *β*-cell proliferation?

### Weaning-dependent loss of milk miRNAs: the maturation signal for *β*-cells

Exosomal miRNA traffic plays an important role in *β*-cell regulation, especially cell-to-cell communication between *β*-cells as well as *β*-cell cross-talk with circulatory exosomes derived from distant tissues involved in the regulation of glucose homeostasis [58–62]. Notably, exosomes released from lipid-induced insulin-resistant muscles have been shown to modulate miRNA-dependent gene expression and proliferation of recipient *β*-cell cells in mice [61]. Furthermore, pancreatic cancer cells release exosomes into the systemic circulation that readily reach *β*-cells and impair insulin secretion (paraneoplastic diabetes) [63]. Remarkably, the islet capillary network exhibits five times higher density than the capillary network of the exocrine counterpart and shows high permeability [64]. The islet microvascular endothelial cells have about 10 times more fenestrations than those of the exocrine tissue [64]. These *fenestrae* have an unusually extensive pore size of 100 nm in diameter [64] allowing a rapid passage of macromolecules and exosomes most likely including milk-derived exosomes (30–100 nm) [65]. Bovine milk exosomes have been detected in multiple tissues and organs after oral administration to mice [20, 21]. It is thus conceivable that milk-derived exosomes maintain a signaling cross-talk to the *β*-cells of the milk recipient, who physiologically is the newborn infant requiring maternal milk exosomes for adequate mTORC1-dependent *β*-cell proliferation and *β*-cell mass extension associated with suppression of AMPK.

### Milk-derived miRNAs: suppressors of AMPK and activators of mTORC1

**Fig. 1:**
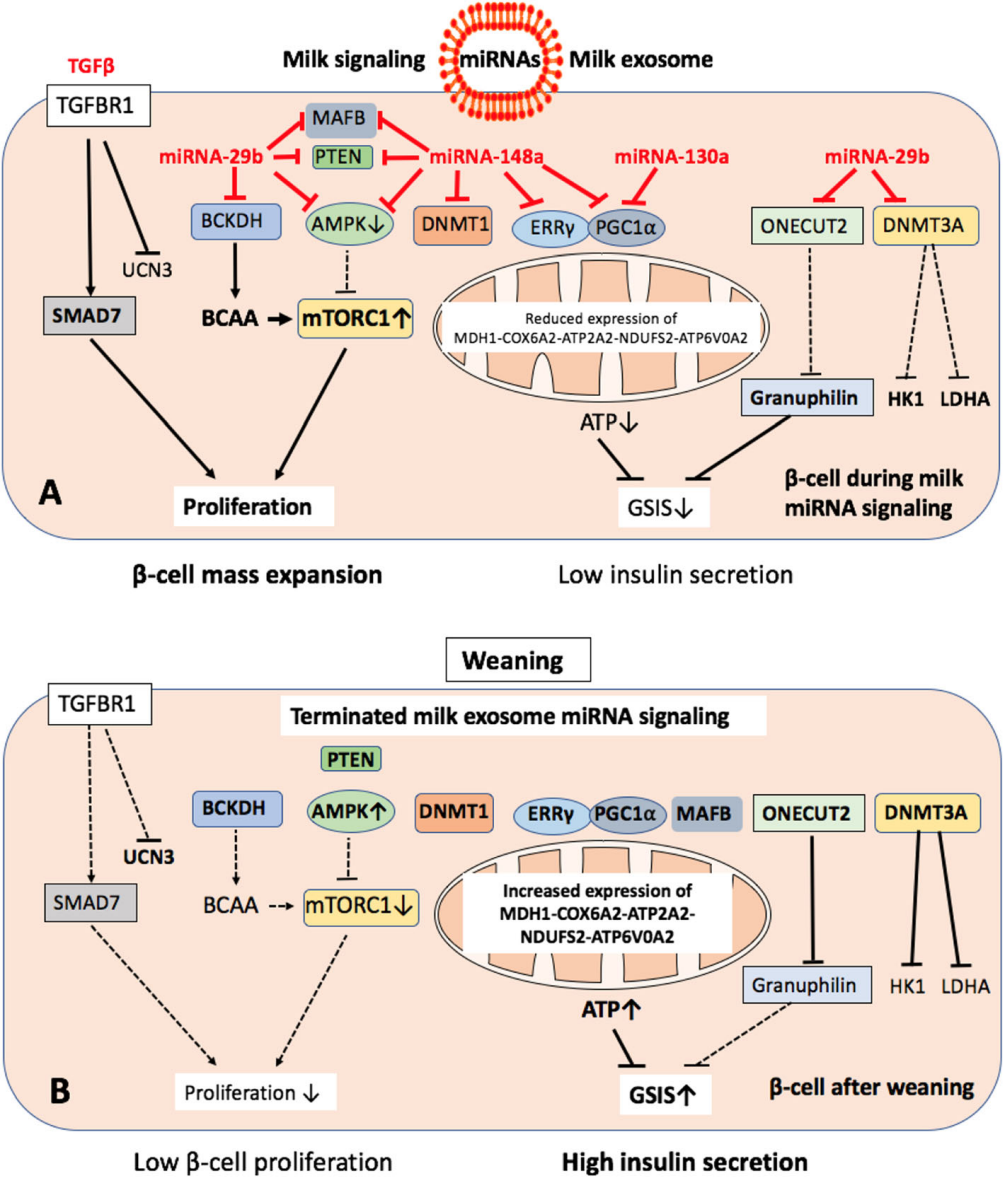
**a** Milk exosome-mediated miRNA signaling of pancreatic *β*-cells during physiologic breastfeeding and persistence of bovine milk-derived miRNA signaling by continued consumption of pasteurized cow’s milk. miRNA-148a suppresses AMPK and PTEN. miRNA-29b inhibits the catabolism of branched-chain amino acids (BCAAs). Reduced AMPK- and PTEN activity combined with increased BCAA levels activate mTORC1, which promotes *β*-cell proliferation and mass expansion. miRNA-148a together with miRNA-130a suppress the transcription factor complex EER *γ* /PGC1 *α*, which controls multiple mitochondrial genes involved in ATP production required for glucose-stimulated insulin secretion (GSIS). miRNA-29b suppresses ONECUT2, an inhibitor of granuphilin resulting in enhanced suppression of GSIS. miRNA-29b suppresses DNMT3A increasing the expression of “forbidden” genes of mature *β*-cells such as HK1 and LDHA. **b** Weaning terminates milk miRNA signaling. The disappearance of miRNA-148a enhances the activity of AMPK and PTEN resulting in increased suppression of mTORC1, whereas AMPK-dependent gene-regulation is upregulated. EER *γ* /PGC1 *α* activates mitochondrial genes involved in ATP production which in combination with ONECUT2-mediated granuphilin suppression enhance GSIS. However, this mature *β*-cell phenotype de-differentiates again by persistent intake of bovine milk exosomes

AMPK phosphorylates the mTORC1 binding partner raptor [48] and tuberous sclerosis protein TSC2, the upstream suppressor of mTORC1 and thereby suppresses mTORC1 [49]. AMPK mediates cellular energy responses to control mTORC1-dependent cell growth and survival [48, 49]. It has recently been demonstrated in breast cancer cells that upregulation of miRNA-148a inhibits the expression of AMPK [66]. In a highly conserved manner with strong binding affinity, miRNA-148a targets the catalytic subunit *α* 1 of AMPK (*PRKAA1*) as well as the AMPK regulatory subunit *γ* 2 (*PRKAG2*) (targetscan.org) (Table 2). Reif et al. recently demonstrated that milk exosome-derived miRNA-148a also suppresses phosphatase and tensin homolog (PTEN), a pivotal inhibitor of the phosphatidylinositol-3 kinase (PI3K)-AKT-mTORC1 signaling pathway [67]. miRNA-29b and miRNA-29c, other two miRNA components of bovine milk [11], target the AMPK subunit *β* 2 (*PRKAB2*) (targetscan.org) (Fig. 1a). Consumption of pasteurized commercial milk increased the levels of miRNA-29b in plasma and blood monocytes [19], whereas bacterial fermentation of milk decreased miRNA-29b concentration [39]. miRNA-29b also targets *DBT* (dihydrolipoamide branched-chain transacylase) [68], the E2 core component of branched-chain *α*-keto acid dehydrogenase, the rate-limiting enzyme of branched-chain amino acid (BCAA) catabolism. BCAAs are key activators of mTORC1 [69–72] and are increased in plasma of patients with T2DM [73–80].

**Table 2:** Potential target genes of exosomal milk miRNA-148a and predicted regulatory effects

miRNA-148a target genes	Transcription factors and enzymes	Expected regulatory effects	Ref.
*PRKAA1*	AMP-activated protein kinase, catalytic subunit *α* 1	Reduced activity of AMPK resulting in activation of mTORC1	[66]
*PRKAG2*	AMPK regulatory subunit *γ* 2	Reduced activity of AMPK resulting in activation of mTORC1	Targetscan.org
*MAFB*	V-MAF musculoaponeurotic fibrosarcoma oncogene family, protein B	Reduced expression of *SCL2A2*, *SCL30A6*, *CAMK2B*, *NNAT* and *MAFA*	[101]
*ESRRG*	Estrogen-related receptor- *γ*	Reduced expression of *MDH1*, *COX6A2*, *ATP2A2*, *NDUFS2* and *ATP6V0A2*	Targetscan.org
*PPARGC1A*	Peroxisome proliferator-activated receptor- *γ*, coactivator 1 *α* (PGC1 *α*)	Reduced expression of *MDH1*, *COX6A2*, *ATP2A2*, *NDUFS2* and *ATP6V0A2*	Targetscan.org
*DNMT1*	DNA methyltransferase 1	Reduced repression of *ARX*	[91, 92]
*PTEN*	Phosphatase and tensin homolog	Increased PI3K-AKT-mTORC1 signaling	[66] Targetscan.org
*WNT1*	Wingless-type MMTV integration site family, member 1	Reduced suppression of adipogenesis	[139] Targetscan.org
*WNT10B*	Wingless-type MMTV integration site family, member 10B	Reduced suppression of adipogenesis	[140] Targetscan.org
*CCKBR*	Cholecytokinin B receptor	Decreased satiety signaling	[146–148] Targetscan.org

It is thus conceivable that weaning-associated termination of milk miRNA-mediated suppression of AMPK, PTEN and DBT decreases *β*-cell mTORC1 activity promoting the metabolic switch to AMPK-regulated *β*-cell maturation with appropriate GSIS (Fig. 1b).

### Milk miRNA-148a: regulator of AMPK-controlled disallowed genes

In the *β*-cell, a small group of genes, which are abundantly expressed in most if not all other mammalian tissues, are highly selectively repressed such as lactate dehydrogenase A (*LDHA*) and monocarboxylate transporter-1 (*SLC16A1*). Their inactivation ensures that pyruvate and lactate, derived from muscle during exercise, do not stimulate inappropriate insulin release [81, 82]. Loss of AMPK from *β*-cells up-regulates the *β*-cell-disallowed gene family resulting in *β*-cell de-differentiation characterized by increased expression of normally repressed (“disallowed”) genes, such as *LDHA*, *SLC16A1*, *MGST1*, *PDGFRA* leading to aberrant fuel sensing [83]. Elevated AMPK activity, which is stimulated by metformin, is required to suppress these disallowed *β*-cell genes [83–85]. Milk-miRNA-148a-mediated suppression of AMPK may thus compromise *β*-cell identity and function to maintain the postnatal immature, proliferating phenotype (Fig. 1a ).

### miRNA-148a: critical suppressor of estrogen-related receptor- *γ*

AMPK activation triggers a switch to oxidative metabolism in mature islets [41]. Yoshihara et al. recently identified estrogen-related receptor- *γ* (ERR *γ*) encoded on *ESRRG* as a master regulator of *β*-cell maturation that is expressed in the adult, but not neonatal β-cell [86]. ERR *γ* is a crucial mediator of multiple endocrine and metabolic signals and plays important roles in *β*-cell maturation [86, 87]. Postnatal induction of ERR *γ* drives a transcriptional network activating mitochondrial oxidative phosphorylation, tricarboxylic acid (TCA) cycle, fatty acid oxidation, the electron transport chain (ETC), and ATP production needed to drive GSIS. Mice deficient in *β*-cell-specific ERR *γ* expression are glucose intolerant and fail to secrete insulin in response to a glucose challenge [86]. During the postnatal period before weaning, ATP-dependent insulin secretion is low [86]. However, during *β*-cell maturation, ERR *γ* upregulates several mitochondrial genes (*MDH1*, *COX6A2*, *ATP2A2*, *NDUFS2*, and *ATP6V0A2*) [87]. Notably, ERR *γ* binds to its co-activator PGC-1 *α* (peroxisome proliferator-activated receptor- *γ* co-activator 1 *α*; *PPARGC1A*) producing a stable transcription factor ERR *γ* /PGC-1 *α* complex [88, 89]. Both mRNAs of *ESRRG* and *PPARGC1A* exhibit highly conserved binding sites (8-mer) for miRNA-148a, miRNA148b and miRNA-152, whereas *PPARGC1A* exhibits further conserved binding sites for miRNA-130a, miRNA-29a, miRNA-29b, and miRNA-29c (targetscan.org) (Fig. 1a). Remarkably, it has been shown that elevated expression of miRNA-130a, miRNA-130b and miRNA-152 suppresses GSIS via modulation of intracellular ATP levels [54]. miRNA-130a is another abundant miRNA of cow’s milk [11]. miRNA-152 is a member of the miRNA-148a/miRNA-148b/miRNA-152 family, which all share identical seed sequences [90]. Thus, termination of miRNA-148a/miRNA-130a/miRNA-29b signaling of milk most likely explains the critical switch to the adult metabolically mature *β*-cell phenotype with adequate GSIS (Fig. 1b). It is conceivable that this pathway may operate in concert with other miRNAs and RNA signaling networks, but due to its abundance in milk, miRNA-148a may play the leading role.

### Cessation of milk miRNA signaling stabilizes *β*-cell identity

DNA methylation directs functional maturation of pancreatic *β*-cells. It has been demonstrated that *β*-cell identity is maintained by DNA methylation-mediated repression of the lineage determination gene *aristaless-related homeobox, X-linked* (*ARX*) [91]. *β*-Cells deficient in DNA methyltransferase 1 (DNMT1), the maintenance DNA methyltransferase that propagates DNA methylation patterns during cell division, were converted to *α*-cells [91]. Propagation of DNA methylation during cell division is essential for repression of *α*-cell lineage determination genes to maintain *β*-cell identity. Milk miRNA-148a, which targets DNMT1 [35, 92], may thus prevent DNMT1-mediated *β*-cell differentiation.

DNMT3A also promotes *β*-cell differentiation. In a murine model, *β*-cell-specific deletion of DNMT3A prevented the metabolic switch, resulting in loss of GSIS [93]. DNMT3A binds to the promoters of the genes encoding hexokinase 1 (*HK1*) and lactate dehydrogenase A (*LDHA*), both of which regulate the metabolic switch. Knockdown of these two key DNMT3A targets restored GSIS response in islets from animals with *β*-cell-specific DNMT3A deletion. Furthermore, DNA methylation-mediated repression of glucose-secretion decoupling genes to modulate GSIS was conserved in human *β*-cells. Remarkably, DNMT3A is a target of miRNA-29b [94–96]. Together, these observations reveal a critical epigenetic role for weaning, i.e., the loss of milk miRNAs, for DNA methylation-mediated *β*-cell maturation (Fig. 1b).

### Milk miRNA-148a: suppressor of β-cell MAFB

The transcription factor MAFB plays a key role in late events essential for *β*-cell maturation and activates genes involved in mature endocrine functions including those significant for glucose sensing (*SCL2A2*), vesicle maturation (*SCL30A6*), Ca^2+^ signaling (*CAMK2B*) and insulin secretion (*NNAT*) [97, 98]. A switch from MAFB to MAFA expression accompanies differentiation to pancreatic *β*-cells [99]. MAFB increases the expression of MAFA, which is important to maintain pancreatic *β*-cell function in adults [97, 100]. MAFB is a direct target of miRNA-148a [101]. miRNA-148a has been detected as a major miRNA component of bovine milk and milk fat [25, 26]. Notably, MAFA was lower in islets of mice that continuously received milk fat [40]. Thus, persistent milk consumption in adults may maintain an immature *β*-cell phenotype with compromised MAFB to MAFA conversion (Table 2).

### Milk miRNA-deficient infant formula and impaired *β*-cell maturation

**Fig. 2:**
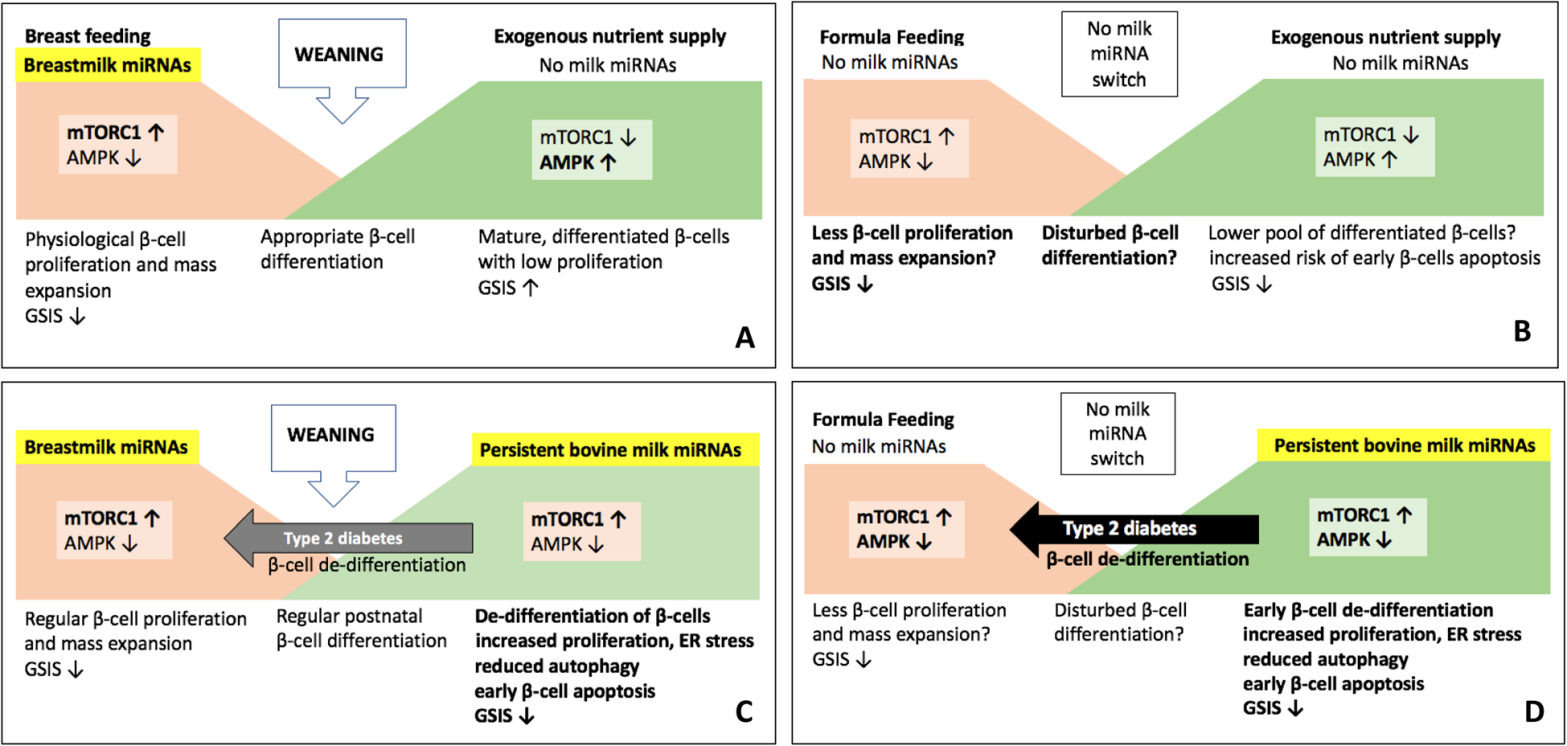
**a** Physiological termination of breastmilk miRNA signaling after weaning. There is an appropriate metabolic switch from mTORC1-driven *β*-cell proliferation and mass expansion to AMPK-driven mature *β*-cell function with GSIS. **b** Infant formula feeding with deficient milk miRNA signaling may impair adequate *β*-cell mass expansion and differentiation to regularly matured *β*-cells. Metabolic challenges of an impaired pool of *β*-cells in later life may enhance the risk of type 2 diabetes. **c** Persistence of milk miRNA signaling after regular breastfeeding by intake of bioactive exosomes of bovine milk may de-differentiate *β*-cell back to a mTORC1 ↑ /AMPK ↓ progenitor phenotype enhancing the risk of type 2 diabetes mellitus. **d** The worst scenario: miRNA-deficient formula-fed infants start into life with a postnatally compromised pool of *β*-cells, have no appropriate miRNA-dependent metabolic switch to mature *β*-cells and may thus experience early de-differentiation of their *β*-cells to the mTORC1 ↑ /AMPK ↓ progenitor phenotype, that increases mTORC1-driven endoplasmic reticulum (ER) stress with reduced autophagy and early *β*-cell apoptosis

There is general agreement that breastfeeding protects against T2DM [102–106]. Human breastmilk and milk lipids contain and transfer bioactive miRNAs including miRNA-148a [26–29, 106, 107]. In contrast, infant formula exhibits severe deficiencies of miRNAs including miRNA-148a (Fig. 2b, d) [108–110]. It is thus conceivable that miRNA-deficient formula feeding in comparison to breastfeeding may impair postnatal *β*-cell proliferation with appropriate acquisition of *β*-cell mass increasing the risk of T2DM later in life. The systemic availability of bioactive miRNAs in breastmilk may explain the superiority of breastfeeding compared to miRNA-deficient formula feeding in the prevention of T2DM. However, the beneficial effect on milk miRNA signaling during the breastfeeding period may turn into an adverse effect, when this miRNA signaling process is not terminated.

### Milk miRNAs: potential drivers of β-cell de-differentiation in type 2 diabetes

The top 20 most abundant miRNAs are shared between mammalian species including humans, especially miRNA-148a and members of the let-7 family, which points to their key regulatory functions [111]. It has recently been demonstrated that whole blood levels of miRNA-148a, miRNA-122, miRNA-144, miRNA-589, and let-7a were associated with glycemic status [112]. In addition, miR-148a and miRNA-144 were associated with elevated glucose levels, and miRNA-148a and other miRNAs were associated with HbA1c levels [112]. These data underline the diabetogenic role of miRNA-148a, which represents the most abundant miRNA species of human and bovine milk exosomes and milk fat [25–29]. Accumulating evidence suggests that a functional deficiency, involving de-differentiation of the mature *β*-cell towards a more progenitor-like state, may be an important driver for impaired insulin secretion in T2DM [113]. In fact, Jaafar et al. provided recent evidence that T2DM, a condition marked by both mitochondrial degeneration and dysregulated GSIS, was associated with a remarkable reversion of the normal AMPK-dependent adult *β*-cell signature to a more neonatal one characterized by mTORC1 activation (Fig. 2a, b) [41]. Persistently over-activated mTORC1 has been associated with increased endoplasmic reticulum (ER) stress and impaired autophagy resulting in early *β*-cell apoptosis [70, 71, 114–120].

Persistent consumption of pasteurized milk is apparently the ideal endocrine signaling mechanism that maintains mTORC1 over-activation combined with milk-miRNA-148a/miRNA-29b-mediated suppression of AMPK, ERR *γ*, DNMT1, DNMT3A and MAFB promoting both mTORC1-dependent *β*-cell stress as well as *β*-cell de-differentiation with functional impairments of GSIS. These observations shed a new light on milk exosomal miRNAs operating as critical environmental promoters of T2DM, i.e., milk-derived diabetogenic miRNA toxicity.

### Milk transforming growth factor- *β* in *β*-cell de-differentiation

Functional *β*-cell maturation is marked by an increased glucose threshold and by expression of urocortin 3 (*UCN3*) [121]. UCN3 is expressed in adult *β*-cells in both mouse and humans and appears late in *β*-cell differentiation [122]. Loss of UCN3 expression is an early event in β cell de-differentiation in T2DM [123]. A small molecule inhibitor of TGF-β receptor 1 (*TGFBR1*) has been shown to protect *β*-cells from the loss of key *β*-cell transcription factors and restores a mature *β*-cell identity including UCN3 expression even after exposure to prolonged and severe T2DM. Inhibition of TGF- *β* receptor 1 was found to protect against *β*-cell de-differentiation and to restore the identity of mature *β*-cells. Notably, exosomes of commercial milk contain and transfer bioactive TGF- *β* to the milk recipient [124]. Upregulation of SMAD7, a downstream mediator of TGF- *β* signaling, promotes *β*-cell proliferation [125]. Milk exosome-mediated TGF- *β* signaling in synergy with milk miRNAs may thus promote proliferation and de-differentiation of *β*-cells (Fig. 1a).

### Milk miRNA-29: insulin resistance and disturbed energy homeostasis

The preferred uptake of milk exosomes by the liver may enhance hepatic miRNA-29b levels [20, 23]. Aberrantly enhanced expression of miRNA-29b has been reported across five common rodent models of insulin resistance (IR) and diabetes (OB = leptin-deficient ob/ob mice; STZ = streptozotocin-treated mice; ZF = fa/fa Zucker Fatty rats; UCD = rat model of late-onset type diabetes (UC Davis); LIRKO = liver-specific insulin receptor knock-out mice; NOD mice) [126–130]. In skeletal muscle of diabetic Goto-Kakizaki rats, miRNA-29a, miRNAR-29b, and miRNA-29c were significantly upregulated [131]. miRNA-29a and miRNA-29c are increased in skeletal muscle from patients with T2DM and are decreased following endurance training in healthy young men and in rats [132]. miRNA-29b via its seed sequence ACCACGA targets key players of IR such as insulin receptor substrate-1 (IRS1) (Table 3). Hung et al. recently demonstrated that acute suppression of IR-associated hepatic miRNA-29 in vivo using locked nucleic acid (LNA) technology improved glycemic control in adult mice [133]. miRNA-29 suppression resulted in increased expression miRNA-29b targets DNMT3A and ENHO (Table 3). As outlined, DNMT3A is important for *β*-cell differentiation [93], whereas ENHO encodes the energy homeostasis hormone adropin. Remarkably, decreased serum adropin levels have been detected in T2DM patients that negatively correlate with body mass index (BMI) [134]. Adropin deficiency worsens high-fat diet-induced metabolic defects [135]. Milk-miRNA-29b-mediated suppression of adropin may thus increase BMI, a desired mechanism for the growing infant but not intended for body mass homeostasis in adulthood. Milk-mediated transfer of miRNA-29b may thus aggravate both IR and BMI. In fact, IR has been demonstrated in 8-year-old boys consuming 53 g protein daily provided as 1.5 L skim milk compared to boys receiving 53 g protein provided as low-fat meat [3]. In accordance, the NHANES 1999–2004 study identified a correlation between milk consumption and BMI in children [136].

**Table 3:** Highly conserved miRNA-29b target genes involved in the regulation of insulin, glucose and energy homeostasis and branched-chain amino acid metabolism

Gene	Expressed protein	Seed sequence
*IGF1*	Insulin-like growth factor 1	ACCACGA
*IRS1*	Insulin receptor substrate 1	ACCACGA
*PIK3R1*	Phosphatidylinositol 3-kinase regulatory subunit 1	ACCACGA
*PTEN*	Phosphatase and tensin homolog	ACCACGA
*ENHO*	Energy-homeostasis associated protein	ACCACGA
*FOS*	V-FOS FBJ murine osteosarcoma viral oncogene homolog	ACCACGA
*VEGFA*	Vascular endothelial growth factor A	ACCACGA
*DNMT3A*	DNA methyltransferase 3A	ACCACGA
*SPARC*	Secreted protein, acidic, cysteine-rich	ACCACGA
*DBT*	Dihydrolipoamide branched-chain transacylase	ACCACGA
*PRKAB2*	AMP-activated kinase subunit B2	ACCACGA
*SLC16A1*	Monocarboxylic acid transporter 1	ACCACGA
*ONECUT2*	One cut homeobox 2	ACCACGA
*MCL1*	Myeloid cell leukemia sequence 1	ACCACGA
*PPARGC1A*	Peroxisome proliferator-activated receptor- *γ*, co-activator 1 *α*	ACCACGA
*MAFB*	V-MAF musculoaponeurotic fibrosarcoma oncogene family, protein B	ACCACGA

### miRNA-29b and *β*-cell exocytosis

Overexpression of miRNA-29a/b/c in the MIN6 *β*-cell line and dissociated islet cells led to impairment in GSIS [137]. Defective insulin release was associated with diminished expression of the transcription factor ONECUT2, and a consequent rise of granuphilin, an inhibitor of *β*-cell exocytosis. ONECUT2 binds to the granuphilin promoter and represses its transcriptional activity (Fig. 1a) [137]. Silencing of ONECUT2 mimicked the effects of miRNA-9 on stimulus-induced exocytosis and on granuphilin expression [137]. Notably, *ONECUT2* is a target gene of miRNA-29a/b/c [138, targetscan.org] (Table 3). Milk-miRNA-29 may thus attenuate insulin secretion during the physiologically restricted period of breastfeeding.

### Milk miRNA-148a synergizes with high-fat diet-induced adipogenesis

T2DM and obesity (diabesity) with associated IR are intimately related. miRNA-148a is increased in adipose tissues from obese individuals and mice fed a high-fat diet (HFD) [138]. miRNA-148a suppresses its target gene WNT1, an endogenous inhibitor of adipogenesis [139, 140]. Ectopic expression of miRNA-148a, a potential constellation evoked by milk miRNA intake, accelerates differentiation and partially rescued WNT1-mediated inhibition of adipogenesis, whereas knockdown of miRNA-148a inhibited adipogenesis [138, 139]. miRNA-148a also silences WNT10b, a further endogenous inhibitor of adipogenesis [140]. Furthermore, increased expression of miRNA-148a via suppression of DNMT1 enhanced adipocyte differentiation, whereas in the absence of DNMT1 adipocyte-specific gene expression and lipid accumulation occurred precociously [141]. DNA methylation biphasically regulates 3 T3-L1 preadipocyte differentiation [142]. Inhibition of DNA methylation at late stage of preadipocyte differentiation promotes lipogenesis and the adipocyte phenotype in 3 T3-L1 cells, which may be mediated by induction of sterol regulatory element-binding transcription factor 1c (SREBF1c), whose promoter activity is upregulated by DNA demethylation during adipogenesis [143]. Persistent transfer of milk exosomal miRNA-148a may thus enhance SREBF1c-mediated lipid accumulation in adipocytes. In accordance, the *MIR148A* gene has been identified as an obesity risk gene in humans exhibiting single nucleotide polymorphisms which enhance miRNA-148a expression [144, 145].

Furthermore, milk exosome-derived miRNA-148a may induce a state of hyperphagia, which is meaningful for the growing infant during the anabolic time of breastfeeding. The accumulation of milk exosomes in the brain may allow miRNA-mediated fine-tuning of hypothalamic centers regulating satiety control [20, 33]. Cholecystokinin (CCK), released by duodenal I-cell during intestinal nutrient abundance, is an important hormone that induces satiety signals in the hypothalamus via binding to CCK receptor 2 (CCKBR). Remarkably, CCKBR is a direct target of miR-148a [146]. CCKBR deletion was associated with increased body weight and hypothalamic neuropeptide Y (NPY) content, which explains the increased food intake in CCK2R knockout mice [147, 148]. Thus, persistent intake of milk exosomes may directly promote adipogenesis and indirectly maintain a “hungry brain”, synergistic mechanisms promoting diabesity and increased BMI.

### Environmental risk factors promoting milk-miRNA-driven type 2 diabetes

When Neolithic humans took milk from other mammalian species as a nutrient source about 10,000 years ago, the majority of collected milk was processed by natural microbial fermentation, which degrades milk exosomes and their miRNAs [39]. However, with the introduction of pasteurization (78 ° C), bioactive exosomal miRNAs of milk survived and reached the human food chain by refrigerated storage since the 1950s´. Remarkably, since that time, T2DM prevalence increased progressively from 1% to more than 8.5% today with a prognosis of 10% in the near future [149, 150]. Pasteurization and cooling technologies are not the only changes that increase diabetogenic miRNA exposure. Importantly, *MIR148A* has been identified as a domestication gene of dairy cattle that together with *MIR29B1* increases milk yield [151–153]. Genetic selection for high performance dairy cows may thus has increased the miRNA-related diabetogenic toxicity of commercial milk [32, 33].

## Conclusions

Presented translational evidence sheds a new light on the physiological role of milk signaling in postnatal and adult regulation of *β*-cell homeostasis. Milk-derived exosomes and their cargo, especially miRNA-148a, miRNA-29b, miRNA-29c, miRNA-130a and TGF- *β*, altogether suppress *β*-cell differentiation and insulin secretion promoting mTORC1-dependent *β*-cell proliferation during the postnatal growth period of *β*-cells. Weaning can be regarded as a physiological “turn-off” signal restricting maternal exosomal miRNA signaling. Accumulated translational evidence supports the hypothesis that the termination of exosomal milk miRNA signaling during weaning presents the appropriate signals for AMPK/DNMT1/DNMT3A-driven *β*-cell maturation towards the adult GSIS phenotype. However, unnatural persistence of milk signaling by continued consumption of pasteurized cow’s milk maintains the immature postnatal progenitor-like state of *β*-cells promoting *β*-cell de-differentiation with over-activation of mTORC1 and suppression of AMPK. Persistently over-activated mTORC1 and insufficient secretory function of milk-miRNA-dedifferentiated *β*-cells finally enhances ER stress, impairs autophagy and promotes early *β*-cell apoptosis explaining the epidemiological link between milk consumption and T2DM (Fig. 2c, d). It should be kept in mind that milk-driven miRNA-148a/miRNA-29b signaling is not the only pathway controlling the balance of mTORC1 and AMPK. Other RNAs may act in concert for adjustments of mTORC1 and AMPK activity. Especially long noncoding RNAs (lncRNAs) are in the focus of RNA research in T2DM [154]. Notably, lncRNAs have recently been detected in bovine milk exosomes and have been shown to be stable during in vitro digestion [155].

In contrast to persistent milk miRNA signaling in adulthood, the absence of milk miRNAs in artificial infant formula may impair regular early postnatal *β*-cell development increasing the risk of T2DM, a plausible new explanation for the diabetes-preventive effect of breastfeeding (Fig. 2c, d) [32, 33].

Before new treatment options for T2DM with exosomes and extracellular vesicles are employed [62, 156], milk’s physiological functions in maternal-neonatal *β*-cell communication should be studied in more detail. The environmental exposure of the human milk consumer to bioactive bovine miRNAs that apparently compromise *β*-cell homeostasis has to be terminated. Milk miRNAs are potential diabetogenic biohazards that should not reach the human food chain [32, 33, 37].

Accumulated evidence allows to conclude that milk-derived exosomes of pasteurized milk represent critical pathogens of Western civilization promoting T2DM and explain the epidemic diabetes as a communicable milk-exosome-mediated disease.

Boiling, ultra-heat-treatment, ultra-sonication and fermentation of milk are effective and required methods to reduce the diabetogenic effects of this common food component of developed societies. Among the known roles of gluco- and lipotoxicity of Western diet, presented translational evidence identified milk miRNA toxicity as a new preventable factor in the pathogenesis of T2DM.

## Data Availability

All data generated or analyzed during this study are included in this published article.

## Abbreviations

AMPK: 5′-adenosine monophosphate-activated protein kinase
ARX: aristaless-related homeobox, X-linked
BCAA: branched-chain amino acid
BCKA: branched-chain α-keto acid
BCKD: branched-chain α-keto acid dehydrogenase
DBT: dihydrolipoamide branched-chain acyltransferase
DNMT1: DNA methyltransferase 1
EPIC: European Prospective Investigation into Cancer and Nutrition
ER: endoplasmic reticulum
ERRγ: estrogen-related receptor-γ
ETC: electron transport chain
EV: extracellular vesicle
GSIS: glucose-stimulated insulin secretion
HK1: hexokinase 1
HPFS: Health Professionals Follow-up Study
IR: insulin resistance
IRS-1: insulin receptor substrate 1
LNA: locked nucleic acid
lncRNA: long noncoding RNA
MAFA: V-MAF avian musculoaponeurotic fibrosarcoma oncogene homolog A
MAFB: V-MAF musculoaponeurotic fibrosarcoma oncogene family, protein B
miRNA: micro-ribonucleic acid
MONICA: monitoring trends and determinants in cardiovascular diseases
mRNA: messenger ribonucleic acid
mTORC1: mechanistic target of rapamycin complex 1
NHS: Nurses’ Health Study
PI3K: phosphatdylinositol-3 kinase
PPARGC1A: peroxisome proliferator-activated receptor- *γ*, coactivator 1 *α* (PGC1 *α*)
PRKAA1: AMP-activated protein kinase, catalytic subunit *α* 1
PTEN: phosphatase and tensin homolog
T2DM: type 2 diabetes mellitus
TCA: tricarboxylic acid
TGFBR1: TGF-β receptor 1
UCN3: urocortin 3
WNT1: wingless-type MMTV integration site family, member 1
WNT10B: wingless-type MMTV integration site family, member 10B;
